# Lateral rectus metastasis from an occult systemic malignancy masquerading as abducens palsy: a case report

**DOI:** 10.1186/1752-1947-2-194

**Published:** 2008-06-05

**Authors:** Mohammad T Masoud, Ajmal Rehman, Yusuf Shaikh

**Affiliations:** 1 Department of Ophthalmology, Cheltenham General Hospital, Cheltenham, GL53 7AG, UK; 2 Department of Ophthalmology, Stirling Royal Infirmary, Stirling, UK; 3 Department of Ophthalmology, Queen's Medical Centre, Nottingham, UK

## Abstract

**Introduction:**

Abduction deficit in the elderly is commonly caused by sixth cranial nerve palsy due to microvasculopathy. However, not all such cases are of neurogenic origin, as our case report shows.

**Case presentation:**

We present the case of a 75-year-old woman who was generally unwell, developed acute diplopia and was found to have a right abduction deficit in a quiet eye with no gross orbital signs and symptoms. A computed tomography scan of the head and orbits revealed a metastatic mass in the right lateral rectus muscle. Systemic evaluation confirmed widespread thoracic and abdominal metastases from an occult systemic malignancy. Lateral rectus metastasis from an occult systemic malignancy was masquerading as abducens palsy.

**Conclusion:**

Orbital metastasis involving extraocular muscles can present as isolated diplopia with minimal local signs and the absence of a history of systemic malignancy. A detailed history and systemic examination can identify suspicious cases, which should be investigated further. The clinician should avoid presuming that such an abduction deficit in the elderly is a benign neurogenic palsy.

## Introduction

Binocular diplopia from an abduction deficit is commonly caused by sixth cranial nerve palsy as a result of microvasculopathy. This is a relatively benign condition that usually improves spontaneously. However, there are conditions with high morbidity and mortality that can masquerade as abducens palsy, as our case shows.

## Case presentation

A 75-year-old woman with a 1-week history of diplopia was referred to the ophthalmology clinic from the medical ward, where she was being treated for atypical pneumonia. She had been unwell for a few months with loss of appetite and weight. Sputum acid-fast bacillus smear and culture tests for tuberculosis were negative. There were no specific symptoms or signs suggestive of giant cell arteritis (GCA) but the erythrocyte sedimentation rate (ESR) was 98 mm/hour and the C-reactive protein was more than 139 mg/dl, which prompted the physicians to arrange a temporal artery biopsy. This was later reported as normal.

**Figure 1:**
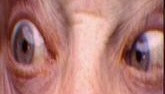
Limitation of right abduction.

Ophthalmic examination showed corrected visual acuity of 6/6 bilaterally. There was subtle localized episcleral injection near the right lateral rectus muscle insertion. The posterior segment revealed a normal optic disc and macula bilaterally. Ocular motility revealed an abduction deficit in the right eye (Figure 1).

**Figure 2:**
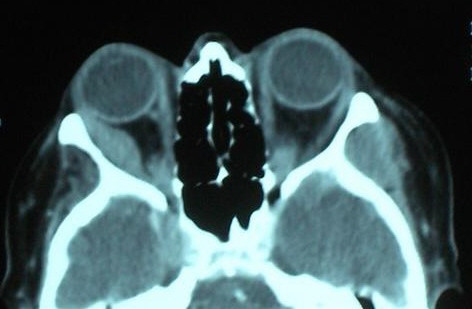
Axial computed tomography scan of the patient showing a mass in the right lateral rectus.

**Figure 3:**
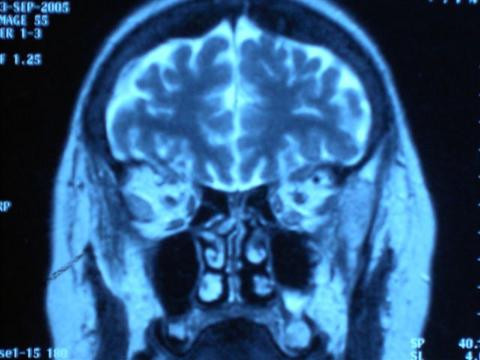
Coronal computed tomography of the patient showing the same mass in the right lateral rectus.

Computed tomography (CT) scanning of the head and orbits revealed a mass in the belly of the right lateral rectus suggestive of a metastatic lesion (Figures 2 and 3). Subsequent CT and magnetic resonance imaging (MRI) of the thorax and abdomen showed multiple secondary metastatic lesions in the lung and enlargement of retroperitoneal lymph nodes. The patient underwent an endoscopic retrograde cholangiopancreatography and the cytology of the brushings was highly suggestive of anaplasia. Pancreatic carcinoma was suspected but abdominal CT scanning and ultrasonic studies revealed no evidence. The patient later developed a supraclavicular lymph node mass, bilateral axillary lymphadenopathy and enlarged spleen. A supraclavicular lymph node biopsy was inconclusive. The suspected diagnosis of pancreatic cancer remained unconfirmed. The general condition of the patient had deteriorated, precluding further invasive investigations such as orbital biopsy. The patient died 4 months after her initial diagnosis of orbital metastasis. Autopsy was not performed on the body.

## Discussion

The orbit is a rare site of distant metastases with incidence varying from 1% to 13% in the reported series of all orbital tumours [1]. The primary malignancies are commonly breast, prostate and lungs and less commonly gastrointestinal tract, kidney, skin (melanoma), thyroid, liver, pancreas, adrenal and salivary glands and choroidal melanoma [1-3].

The diagnosis of orbital metastasis is often unexpected [3] when an ophthalmic manifestation is the first presentation before the primary neoplasm is discovered. This is more frequently seen with lung, gastrointestinal, thyroid and renal carcinomas [3,4]. In contrast, the majority (90%) of breast cancers with ocular metastases have had treatment for the primary tumour before the eye becomes involved [4,5]. In up to 35% of cases, the primary neoplasm remains unknown despite systemic evaluation or autopsy [1-3,6,7]. Most metastatic carcinomas of unknown origin are from the lung or pancreas [6].

The main symptoms of orbital metastasis at presentation include diplopia (48%), proptosis (26%) and decreased vision (16%) [7]. Diplopia is due to either direct tumour infiltration of the muscle or a mass effect; rarely, it is attributed to a paraneoplastic event such as seen with lung carcinoma [8]. Approximately 5% of orbital metastases involve extraocular muscles [9] and they are usually unilateral, unlike choroidal metastases [10]. Devron et al. report no difference between CT and MRI data in establishing the diagnosis of orbital metastases [7]. However, it is important to obtain a brain MRI with gadolinium as these patients may have silent brain lesions when they present with orbital disease. This is helpful in planning radiotherapy [7].

The definitive diagnosis of orbital metastasis can be established with either fine needle aspiration biopsy or an open biopsy. We were unfortunately unable to perform biopsy and hence obtain histological diagnosis due to our patient's deteriorating health.

The prognosis in patients with orbital metastases is often poor as the primary tumour can only be found in one-third of patients [3]. The median survival is reported as a little over 1 year; only 27% have a 2-year survival [3,4]. Management is based on establishing a correct diagnosis, the systemic status of the patient and whether optic nerve compression is present [7].

When dealing with a patient with an abduction deficit, a logical and systemic approach based on its causes is required. Sixth cranial nerve palsy is the most common cause of abduction deficit but myogenic and/or orbital conditions such as dysthyroid eye disease, idiopathic orbital inflammation, muscle entrapment (after blow-out fracture) and orbital metastasis must also be kept in mind [11]. Myasthenia gravis can also mimic any ocular motility defect. Evaluation of isolated, unilateral, non-traumatic abducens palsy depends on the age of the patient. In the elderly population, an isolated sixth nerve paresis is likely to be ischaemic in aetiology and run a benign course. In younger patients, trauma, tumours, demyelination and raised intracranial pressure are important causes.

It cannot be assumed that all cases of abduction deficit are of neurogenic aetiology. A minimal work-up in elderly patients should include blood pressure check, blood glucose and an ESR test. A raised ESR points towards GCA, a treatable condition that can cause extraocular muscle paresis by affecting either the ocular cranial nerves or the extraocular muscles. A detailed history and thorough general physical examination can prove invaluable, especially in atypical cases. Scans of the brain and orbits are not recommended as routine, but should be requested in suspicious cases. It is good practice to liaise with a radiologist and neurologist in such cases. In our patient, the history of prolonged illness accompanied by weight loss, increased ESR and negative temporal artery biopsy raised our suspicions and led to subsequent investigations.

Myogenic and neuromuscular causes for extraocular muscle palsies are not very common and are easily missed. Metastatic muscle involvement, which is rarely described in the ophthalmic literature as a cause of such palsies, needs to be included in the differential diagnoses in suspected cases.

## Conclusion

We can draw the following conclusions:

1. A paretic abduction deficit should not invariably be assumed to be of neurogenic origin. The danger of assuming abduction deficit as a benign ischaemic sixth nerve palsy is especially high in elderly patients.

2. Metastatic orbital tumours are relatively rare but must be included in the differential diagnoses in suspicious cases.

3. Orbital metastasis involving extraocular muscles can present as isolated diplopia in the absence of a history of systemic malignancy.

4. Local ophthalmic signs may be subtle or minimal in orbital and/or myogenic causes of such palsies.

5. It is important to communicate with radiologists when requesting imaging so as to ensure the scans include all areas of interest, namely bothorbits and the brain, in cases of suspected orbital metastasis.

## Abbreviations

CT: computed tomography; ESR: erythrocyte sedimentation rate; GCA: giant cell arteritis; MRI: magnetic resonance imaging.

## Competing interests

The authors declare that they have no competing interests.

## Authors' contributions

MTM, AR and MYS were all involved in the care of this patient. MTM and AR co-wrote the report. As the senior clinician, MYS supervised the work. All authors read and approved the final manuscript.

## Consent

Written informed consent was obtained from the patient for publication of this case report and any accompanying images. A copy of the written consent is available for review by the Editor-in-Chief of this journal.
